# Diagnostic Challenges of LRP4 Antibody Testing in Myasthenia Gravis: A Case Series of Four LRP4 Positive Cases With Uncertain Diagnostic Significance

**DOI:** 10.1155/crnm/3092521

**Published:** 2026-04-30

**Authors:** Katherine Rosella Kinnard, Ehtesham Khalid

**Affiliations:** ^1^ Ochsner Medical Center, New Orleans, Louisiana, USA, ochsner.org; ^2^ Neuromuscular Medicine, Ochsner Medical Center, New Orleans, Louisiana, USA, ochsner.org; ^3^ University of Queensland, Brisbane, Australia, uq.edu.au

**Keywords:** AChR antibody, LRP4 antibody, MuSK antibody, myasthenia gravis

## Abstract

Myasthenia gravis is an autoimmune disorder characterized by muscle weakness due to impaired neuromuscular transmission. While antibodies against the acetylcholine receptor and muscle‐specific kinase are commonly used for diagnosis, a subset of patients remains seronegative, necessitating alternative biomarkers. Low‐density lipoprotein receptor‐related Protein 4 antibodies have emerged as a potential diagnostic marker in seronegative myasthenia gravis cases. However, misleading positive results have obscured diagnosis and management. This study explores the clinical relevance of low‐density lipoprotein receptor‐related Protein 4 antibodies in myasthenia gravis through four patient case studies. Patients presenting with concern for myasthenia gravis underwent a comprehensive diagnostic evaluation, including antibody testing for acetylcholine receptor, muscle‐specific kinase, and low‐density lipoprotein receptor‐related Protein 4. Volitional single‐fiber electromyography was also performed. All tests were employed per standardized laboratory protocols. All four patients were initially evaluated for suspected myasthenia gravis; however, despite positive low‐density lipoprotein receptor‐related Protein 4 antibody results, comprehensive clinical and electrophysiological evaluation ultimately excluded the diagnosis of myasthenia gravis. These cases underscore the limitations of low‐density lipoprotein receptor‐related Protein 4 antibody testing.

## 1. Introduction

As the most common neuromuscular junction (NMJ) disorder, myasthenia gravis (MG) is characterized by impaired signal transmission at the NMJ and manifests as fatigable weakness in the limb, bulbar, and ocular muscles [1–3]. MG is an autoimmune disorder, whereby autoantibodies target key components of the NMJ such as the acetylcholine receptor (AChR) and muscle‐specific kinase (MuSK). Serum levels of these antibodies have been long‐standing primary biomarkers for MG. However, approximately 10% of patients are seronegative for AChR and MuSK antibodies, complicating the diagnosis [4]. Patients within this subgroup responded to standard immune therapies, pointing toward the existence of other autoimmune etiologies to explain their symptoms. This offers the dilemma of identifying alternative biomarkers to improve diagnostic accuracy in seronegative patients with MG. Low‐density lipoprotein receptor‐related Protein 4 (LRP4) antibodies may be observed in seronegative patients, but the particulars of these antibodies are underdeveloped [4, 5].

A NMJ is a synaptic connection between a motor neuron and the muscle fiber it innervates, and its understanding has provided insights into the synaptic process and disorders such as MG. It has been hypothesized that LRP4 plays a vital role in NMJ formation and maintenance [6]. Upon binding agrin from motor neurons, LRP4 activates the receptor tyrosine kinase MuSK, ultimately aggregating AChR at the NMJ. This suggests that LRP4 autoantibodies damage the NMJ by interfering with agrin‐musk signaling, thereby inhibiting agrin‐induced AChR clustering [5–8].

For seronegative MG patients, LRP4 antibodies have been proposed as an additional biomarker [5, 7]. However, the diagnostic reliability remains a concern due to a stunted understanding of their pathophysiological mechanisms and role in MG. This case series highlights four patients presenting with MG‐like symptoms where LRP4 autoantibodies were tested and yielded false‐positive results, emphasizing the need for comprehensive evaluation.

**FIGURE 1:**
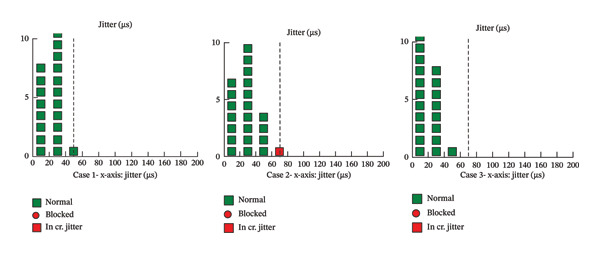
Representative sfEMG results from patients with false‐positive LRP4 antibody results. Despite serological positivity for LRP4 antibodies, the sfEMG results were either within normal limits or showed nonspecific findings inconsistent with NMJ impairment. These images underscore the importance of electrophysiological testing in confirming or excluding the diagnosis of MG in cases with ambiguous serological results. Notably, sfEMG data for Case 4 were unavailable, as the test was performed at an outside facility. Case 1: sfEMG jitter values for Case 1 demonstrated normal transmission, with all recorded potentials falling within expected limits and no evidence of increased jitter or blocking. Case 2: sfEMG in Case 2 showed predominantly normal jitter values with only a single potential demonstrating mild, nonspecific abnormality. No sustained increased jitter or blocking was observed. Case 3: sfEMG in Case 3 exhibited jitter values within normal limits, with no increased jitter or blocking identified.

This case series involves a retrospective analysis of patients ranging from 2016 to 2024 referred for evaluation of LRP4‐positive suspected MG at the Ochsner Clinic Foundation. All patients underwent comprehensive diagnostic testing, including serological, clinical, and volitional single‐fiber electromyography (sfEMG). Volitional sfEMG was performed according to the current national standards, with assessment of mean consecutive difference (jitter) and blocking using established age‐adjusted reference values. A study was considered abnormal if jitter exceeded published normative limits or if abnormal blocking was present. Serologic studies included reflex testing for AChR and MuSK antibodies. Additional laboratory evaluation included complete blood count (CBC), comprehensive metabolic panel (CMP), thyroid function testing, and other ancillary studies to exclude metabolic or systemic contributors to weakness. Repetitive nerve stimulation was not performed in these patients, as sfEMG was utilized as the primary electrophysiologic modality due to its sensitivity for detecting NMJ transmission defects [9]. Muscle biopsy was not performed on this set of patients. Patient data (including demographic information), presenting symptoms, serological results, electrophysiological findings, and final diagnoses were systematically recorded via chart review and managed using an Excel spreadsheet. LRP4 antibody testing was performed by Athena Diagnostics (Marlborough, Massachusetts) using an indirect immunofluorescence assay on a recombinant cell line expressing human LRP4 antigen. Results were reported qualitatively as positive or negative according to the laboratory’s internal reference standards. Quantitative titers were not provided. As testing was performed externally, confirmatory in‐house cell‐based assays or alternative platforms were not available. Patients were selected based on clinical symptoms suggestive of MG, negative AChR and MuSK antibody results, and positive LRP4 autoantibody results. Out of 61 patients tested for LRP4 autoantibodies, 5 were found positive. One patient has been excluded from our case series, as they tested positive for MuSK antibodies. The final diagnoses varied widely and are summarized in Figure 1.

## 2. Case Presentation

 Case 1: A 49‐year‐old male with chronic migraines complained of left eye ptosis beginning in February of 2023, which had worsened over time. MG was initially suspected due to unilateral ptosis and facial weakness. There was additional weakness in the left arm and leg. On examination, there was observable ptosis of the left eye but no fatigable weakness. The diagnostic evaluation involved an ice pack test, sfEMG, serologic studies (Table 1). Magnetic resonance imaging (MRI) of the brain was negative for any acute abnormalities. The lack of electrophysiological or serological confirmation ultimately led to the exclusion of MG.
 Case 2: A 74‐year‐old male with a history of alcohol overuse presented with imbalance, decreased mobility, and significant fatigue. He denied diplopia, respiratory distress, or dysphagia. However, MG was considered due to his generalized fatigue and functional decline. Initially, the patient was unable to ambulate without support. He also exhibited a resting and action tremor in the bilateral hands. The diagnostic evaluation involved an ice pack test, sfEMG, and serologic studies (Table 1). MRI of the brain was negative for any acute abnormalities. The lack of electrophysiological or serological confirmation ultimately led to the exclusion of MG.
 Case 3: A 78‐year‐old female presented with intermittent left facial droop and bilateral upper extremity paresthesias. Her presentation was atypical, yet MG was considered given the intermittent facial weakness. The diagnostic evaluation involved an ice pack test, sfEMG, serologic studies (Table 1). MRI of the brain was negative for acute abnormalities. The lack of electrophysiological or serological confirmation ultimately led to the exclusion of MG.
 Case 4: A 19‐year‐old male born prematurely with perinatal injury to the hypoglossal nerve complained of worsening facial weakness, articulation issues, weakness in the hands, and poor exercise tolerance since January 2024. MG was initially considered because of worsening bulbar symptoms in a young adult. The diagnostic evaluation involved an ice pack test, sfEMG, and serologic studies (Table 1). MRI of the brain was negative for any acute abnormalities. The lack of electrophysiological or serological confirmation ultimately led to the exclusion of MG.

**TABLE 1:** Summary of clinical and diagnostic findings in patients with false‐positive LRP4 antibody results.

	Age/gender	Initial symptoms	AChR/MuSK antibodies	LRP4	Repetitive stimulation	sfEMG	Ice pack test
Case 1	49‐year‐old male	Left eye ptosis	Both negative	Positive	Not performed	Negative	Negative
Case 2	74‐year‐old male	Decreased mobility and balance; significant fatigue	Both negative	Positive	Not performed	Equivocal (5% blocking)	Not performed
Case 3	78‐year‐old male	Intermittent left facial droop	Both negative	Positive	Not performed	Negative	Not performed
Case 4	19‐year‐old male	Decreased facial strength and exercise intolerance	Both negative	Positive	Not performed	Negative	Not performed

*Note:* This chart provides an overview of the key characteristics of patients included in the case series. AChR = acetylcholine receptor, MuSK = muscle specific kinase, LRP4 = low‐density lipoprotein receptor‐related Protein 4, and sfEMG = single‐fiber electromyography.

## 3. Discussion

All patients were initially referred for evaluation of suspected MG based on clinical features including ocular or bulbar weakness, fluctuating symptoms, or unexplained fatiguability. Given this concern, a structured diagnostic evaluation for MG was undertaken in each case.

The diagnosis of MG was excluded only after the assessment failed to demonstrate objective evidence of NMJ transmission failure. Specifically, exclusion was based on the absence of reproducible fatigable weakness on serial neurological examinations, negative AChR and MuSK antibody testing, normal or nondiagnostic sfEMG findings without sustained increased jitter or pathological blocking consistent with NMJ impairment, and lack of objective improvement with ice pack testing when performed. Thus, while MG was appropriately considered at presentation, objective diagnostic criteria were not met following full evaluation.

LRP4 antibodies have been proposed as a potential biomarker for seronegative MG, yet their diagnostic utility remains uncertain. In our series, all four patients were initially referred for evaluation of MG based on their presenting symptoms. However, following clinical, serologic, and electrophysiologic assessment, none demonstrated objective evidence of NMJ transmission failure consistent with MG, despite isolated LRP4 positivity. Positive LRP4 autoantibody assays complicate the diagnostic landscape for MG, particularly in patients with nonspecific neuromuscular symptoms or atypical pattern of presentation. The positive LRP4 results in our four cases raise critical questions about the reliability of LRP4 assays and the risk of misdiagnosis for NMJ deficits, including MG. Antibodies in other autoimmune or neuromuscular disorders may cross‐react with LRP4, leading to false antibody positive findings [4, 7, 10].

Interpretation of LRP4 antibody positivity is limited by the absence of standardized reference ranges, inconsistent assay methodologies, and lack of validated sensitivity or specificity. Indirect immunofluorescence staining of LRP4 antibodies on recombinant cell line expressing antigen was used in all our cases. The assay used has known susceptibility to nonspecific binding. Developing standardized assays with clearly defined sensitivity and specificity parameters are crucial. Confirmatory cell‐based assays were unavailable for these patients. Cell‐based assays may offer greater specificity and should be considered for confirmatory testing in case of suspicion for false‐positive results or should be reflexed for confirmation in case of positive results. Large‐scale multicenter studies are needed to validate the clinical utility of LRP4 testing, especially in diverse populations and in comparison with other diagnostic tools [10].

This case series demonstrates the diagnostic complexities posed by false‐positive LRP4 antibody (without electrodiagnostic evidence of neuromuscular junction deficit) results in patients with suspected MG. The author recommends avoiding treatment as myasthenia gravis in the setting of positive LRP4 antibodies without single‐fiber EMG or repetitive stimulation confirmation. While LRP4 may have a potential value in seronegative MG, the current assay limitations significantly restrict their utility. Clinicians should rely on a multimodal diagnostic approach that integrates clinical exam, sfEMG, imaging, and confirmatory testing. Improved standardization and validation of LRP4 antibody assays are critical to enhancing diagnostic accuracy and reducing the risk of false‐positive results. Until such advancements are achieved, clinicians must exercise caution in interpreting LRP4 results and prioritize holistic patient assessment to ensure accurate diagnosis and effective management.

## 4. Limitations

This study has several limitations. The small sample size limits broader applicability. The retrospective design introduces variability in documentation. Confirmatory LRP4 cell‐based assays were not available, reducing the reliability of positivity. Final diagnoses for the full cohort of sixty‐one patients were recorded, but the present study focuses only on the four false‐positive cases.

Several potential confounders may contribute to false‐positive LRP4 results. Low‐titer nonspecific binding has been reported in patients with other autoimmune or inflammatory conditions, and assay‐based cross‐reactivity with unrelated antibodies may occur. Advanced age, systemic illness, alcohol‐related neuropathy, migraine‐associated neurological symptoms, and prior structural neurological injury (as seen in our cohort) may produce clinical features that prompt testing despite lacking true NMJ pathology. Additionally, indirect immunofluorescence assays may be susceptible to background reactivity or subjective interpretation. These factors highlight the importance of interpreting isolated LRP4 positivity within the full clinical and electrophysiologic context.

## Funding

No funding was received for this manuscript.

## Ethics Statement

We confirm that we have read the Journal’s position on issues involved in ethical publication and affirm that this report is consistent with those guidelines.

## Conflicts of Interest

The authors declare no conflicts of interest.

## Data Availability

The data that support the findings of this study are available from the corresponding author upon reasonable request.
